# Angiogenic gene characterization and vessel permeability of dermal microvascular endothelial cells isolated from burn hypertrophic scar

**DOI:** 10.1038/s41598-022-16376-z

**Published:** 2022-07-18

**Authors:** Esteban A. Molina, Brandon Hartmann, Mary A. Oliver, Liam D. Kirkpatrick, John W. Keyloun, Lauren T. Moffatt, Jeffrey W. Shupp, Taryn E. Travis, Bonnie C. Carney

**Affiliations:** 1 Georgetown University School of Medicine, Washington, DC USA; 2 Firefighter’s Burn and Surgical Research Laboratory, MedStar Health Research Institute, 110 Irving Street, NW, GHRB, Room 310, Washington, DC 20010 USA; 3 Howard University College of Medicine, Washington, DC USA; 4 Department of Biochemistry and Molecular and Cellular Biology, Georgetown University School of Medicine, Washington, DC USA; 5 Department of Surgery, Georgetown University School of Medicine, Washington, DC USA; 6 The Burn Center, Department of Surgery, MedStar Washington Hospital Center, Washington, DC USA

**Keywords:** Cell biology, Medical research

## Abstract

Hypertrophic scar (HTS) formation is a common challenge for patients after burn injury. Dermal microvascular endothelial cells (DMVECs) are an understudied cell type in HTS. An increase in angiogenesis and microvessel density can be observed in HTS. Endothelial dysfunction may play a role in scar development. This study aims to generate a functional and expression profile of HTS DMVECs. We hypothesize that transcript and protein-level responses in HTS DMVECs differ from those in normal skin (NS). HTSs were created in red Duroc pigs. DMVECs were isolated using magnetic-activated cell sorting with ulex europaeus agglutinin 1 (UEA-1) lectin. Separate transwell inserts were used to form monolayers of HTS DMVECs and NS DMVECs. Cell injury was induced and permeability was assessed. Gene expression in HTS DMVECS versus NS DMVECs was measured. Select differentially expressed genes were further investigated. HTS had an increased area density of dermal microvasculature compared to NS. HTS DMVECs were 17.59% less permeable than normal DMVECs (*p* < 0.05). After injury, NS DMVECs were 28.4% and HTS DMVECs were 18.8% more permeable than uninjured controls (28.4 ± 4.8 vs 18.8 ± 2.8; *p* = 0.11). PCR array identified 31 differentially expressed genes between HTS and NS DMVECs, of which 10 were upregulated and 21 were downregulated. qRT-PCR and ELISA studies were in accordance with the array. DMVECs expressed a mixed profile of factors that can contribute to and inhibit scar formation. HTS DMVECs have both a discordant response to cellular insults and baseline differences in function, supporting their proposed role in scar pathology. Further investigation of DMVECs is warranted to elucidate their contribution to HTS pathogenesis.

## Introduction

Hypertrophic scar (HTS) formation is a common result of insult to deeper layers of the skin from etiologies such as burns, trauma, and surgery^1^. In addition to being aesthetically disfiguring, HTSs can be pruritic, painful, dyspigmented, erythematous, and psychologically impairing. These pathological scars can affect functionality due to contraction, especially when occurring on the face or across joints.

Although the development of HTS is yet to be completely understood, it seems to be a complex multifactorial process involving genetic, systemic, and local factors such as delayed wound healing, wound depth, and skin tension around scars^2^. Histologically, HTSs show excessive deposition of collagen and extracellular matrix (ECM), a thickened epidermis, a hypercellular dermis, and increased microvasculature^3–5^. Different cell types have been studied and implicated in the etiology of HTS, the two major types being fibroblasts and myofibroblasts, which over-proliferate, resist apoptosis, and deposit abnormally excessive ECM in HTS. Myofibroblasts also have contractile capacity and in the process of fibrosis can be derived from several cell types including resident fibroblasts, fibrocytes, epithelial/endothelial cells undergoing epithelial/endothelial-to-mesenchymal transition, vascular pericytes or hepatic stellate cells^6^. In comparison to fibroblasts and keratinocytes, which are known contributors to HTS-symptomology, dermal microvascular endothelial cells (DMVECs) in HTS are less well-studied. In a recent article summarizing the current cellular and molecular mechanisms of HTS, endothelial cells were absent from review^7^.

The vascular endothelium is a monolayer of cells lining the capillaries, acting as a barrier and functioning as an endocrine organ by releasing growth factors. Endothelial cells are sensitive to their environment and respond to local mechanical stimuli. They are involved in vascular tone, leukocyte adhesion and migration, and angiogenesis^8^. Dysfunctional endothelium and chronic mechanical overload is found to be associated with fibrosis in other organs such as the lungs, kidneys and heart^8^. Endothelial dysfunction has been increasingly studied in the setting of trauma and acute burn injury. In prior work, we found that high levels of circulating syndecan-1 (SDC-1), a component of the glycocalyx that lines the luminal surface of blood vessels and is shed upon injury, is associated with an increased 30-day mortality after burn injury^9^. SDC-1 has been found to be up-regulated in a dose-dependent manner in relation to burn injury severity in a rodent model^10^. Due to the link between acute burn care and the long-term systemic effects of burn injury, it is hypothesized that persistent endothelial dysfunction beyond the acute phase may contribute to the formation of HTS. Hypertension, as well as atherosclerosis in revascularized patients, have been linked to increased hypertrophic and keloid scar severity^2,11,12^. Since hypertension can affect vascular function, endothelial dysfunction is further thought to play a role in scar development.

Young HTSs frequently demonstrate hyperemia, suggesting the involvement of microvessels and endothelial cells. The number of blood vessels present has been found to be increased in the dermis of HTSs compared to that of normal skin^13,14^. Increased microcirculation and perfusion can be visualized in HTSs compared to unburned skin using laser doppler imaging^15^. In contradiction, most of the microvessels in HTSs are observed to be occluded or partially occluded through unknown mechanisms, although one possible contributor that has been reported is an excess of endothelial cells within these blood vessels (although from an unknown source)^4^. The process of endothelial-to-mesenchymal transition (EndoMT) has been described in keloids and fibrotic disorders where endothelial cells transition to a mesenchymal or myofibroblastic phenotype and express mesenchymal cell products such as alpha-smooth muscle actin and type I collagen^16,17^. EndoMT may also play a potential role in the formation of HTS.

The formation of blood vessels through angiogenesis is an essential process during wound healing. However, it is probable that aberrant angiogenesis contributes in part to HTS and interventions that target this process may be useful. A balance between the need for angiogenesis during wound healing vs. a restrictive process to prevent HTS is vital. HTS is sometimes thought of as a benign tumor that is “fed” by this abnormal vasculature. The microenvironment in wounds and in tumors share similarities, which was described as long as 30 years ago, and includes hyperproliferation and decreased apoptosis of cells^18^. The number of occluded vessels have been found to be decreased in HTS after compression therapy, so it is thought that compression therapy works in part by “starving” the scar^5^.

Vascular endothelial cell growth factor (VEGF) plays a prominent role in regulating wound healing and angiogenesis in part by stimulating endothelial cell proliferation, migration, differentiation, and survival^19^. This pro-angiogenic growth factor can be produced by epidermal keratinocytes, mast cells, monocytes/macrophages, and activated fibroblasts after injury^19^. VEGF is increased in the early stages of burn scar development and decreases as the scar matures^20–22^. Drugs such as statins, endostatin, and antibodies against VEGF have been studied in rabbit ear models and show improvement in scar metrics^23–25^. Endostatin is a potent endothelial cell proliferation inhibitor, which inhibits angiogenesis and has also been shown to have an effect on tumor growth and metastasis. Despite these promising preliminary studies in the pre-clinical arena however, these treatments are not currently in use for HTS.

The main treatment that is currently in the armamentarium of burn surgeons to treat vascular abnormalities in HTS are laser and light-based technologies. One such technology is intense-pulse light (IPL) which works by targeting hemoglobin wavelengths (560/590 nm) within the scar, and has been shown to reduce erythema (assessed by patient and observer scar scale scores) to a variable degree depending on the patient population^28^. This technology is predominantly used in patients with low Fitzpatrick skin types (FST) (I–III) due to the risk of photobleaching and skin blistering for patients with high FST (V–VI)^26–28^. In addition, the melanin contained within HTS for patients with skin of color may mask erythema that is otherwise visible in fair-skinned patients. However, we have shown that hyper- and hypo-pigmented HTS in red Duroc pigs contain significantly increased vasculature compared to normal skin even if they do not appear to the eye as erythematous^29^. Therefore, it is likely that all patients could benefit from pharmacologic treatments that target endothelial cells and vasculature that may not be possible with light-based treatments. By targeting the genes and proteins that are known to be up-regulated in HTS, these treatments may also be more efficacious and require fewer interventions.

The role of DMVECs in HTS and its mechanisms of involvement remain unclear. In order to characterize HTS DMVECs, this study used a red Duroc pig model, which develops hypervascular HTS similar to that of human HTS^30^. We hypothesize that DMVECs derived from HTSs differ compared to DMVECs from skin based on transcript- and protein-level expression. Pro-angiogenic markers are further hypothesized to be elevated in DMVECs from HTS. By characterizing DMVECs in HTS, we aim to identify factors that may contribute to HTS formation and that could possibly be exploited therapeutically to intervene in the pathogenesis of HTS.

## Materials and methods

### Animal model

Red Duroc swine were handled according to facility standard operating procedures under the animal care and use program accredited by the Association for Assessment and Accreditation of Laboratory Animal Care International (AAALAC) and Animal Welfare Assurance through the Public Health Service (PHS). All described animal work was reviewed and approved by the MedStar Health Research Institute's Institutional Animal Care and Use Committee (IACUC). This study is reported in accordance with ARRIVE guidelines; all details related to the guidelines are included in Carney et al*.*^31^ Animals were fasted for at least 12 h prior to anesthesia induction. Animals were anesthetized with a combination of ketamine (15–30 mg/kg) and xylazine (1–4 mg/kg) that were delivered intramuscularly and were subsequently intubated, maintained on isoflurane anesthesia (3%-5%), placed on a warming blanket, and ventilated for the remainder of the procedure as previously described^31^. Animals were monitored, with vital signs recorded throughout the procedure and recovery period, and with anesthesia adjusted appropriately based on these metrics.

**Figure 1:**
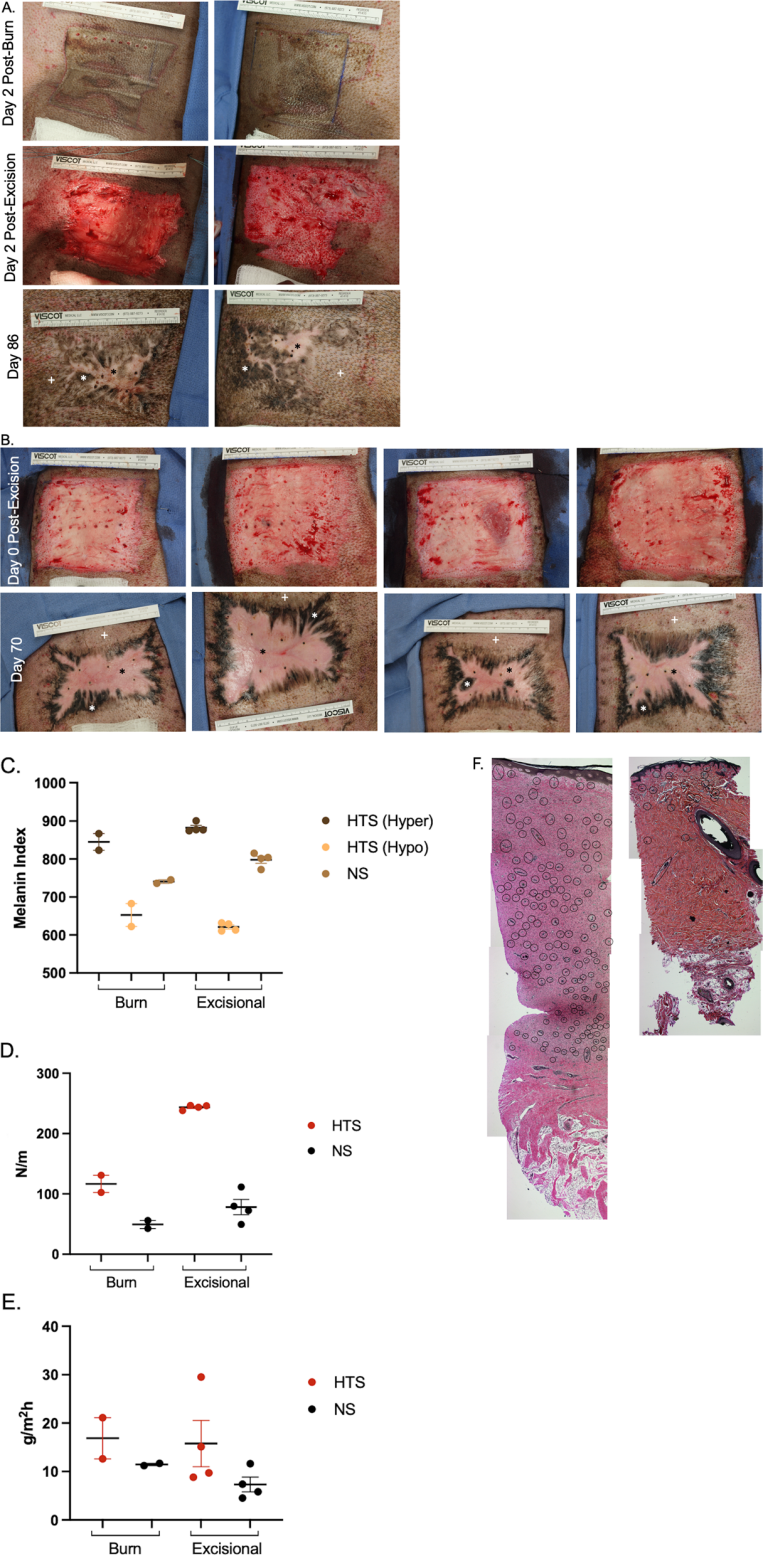
Full thickness burn and injury and excision or excisional injury alone in red Duroc pigs yield HTS with many of the characteristic features of human HTS. Animal 1 burn injury, excision, and final burn HTS (n = 2 different areas) result at Day 86 (**A**). Animal 2 excisional injury and final excisional HTS (n = 4 different areas) result at Day 70 (**B**). Non-invasive measurements of melanin (**C**), elasticity (**D**), and TEWL (**E**). Verhoeff-Van Geison staining of HTS (left) and NS (right) biopsies. Blood vessels (black circles) (**F**). *p* < 0.05*, *p* < 0.01**, *p* < 0.001***, *p* < 0.0001****.

There were two types of injuries that were used to create HTS (Table 1). The first injury type was burn and excisional wound healing (Animal 1, n = 2 scars, Fig. 1A). On Day 0, two full thickness burns were induced in a 10.16 cm × 10.16 cm area with one on each bilateral flank. The burns were created using an aluminum billet set to 150 °C for 10 s of contact with the skin as described previously^31^. These wounds were dressed with Mepilex Ag (Molnlycke, Sweden). On Day 2, the burn wounds were excised using a goulian knife down to bleeding subcutaneous tissue. The wounds were dressed with Mepilex Ag (Molnlycke). Each week through re-epithelialization (~ Day 43), the wounds were cleansed with 1% chlorhexidine gluconate and re-dressed. At Day 84 post-injury, scars were excised along with a (10.14 cm by 10.14 cm) portion of un-injured, normal skin and saved in DMEM culture media with 10% FBS and penicillin/streptomycin at 4 °C until cell isolation occurred. The cells isolated from this injury type were used in permeability assays and PCR arrays.

**Table 1:** Animal injury and experimental usage details.

Animal #	Injury details		Tissue collection	DMVECs used in	N value
1	Full thickness burns (Day 0) (n = 2 HTS)	Burns excised (Day 2)	Day 86	TEER	11 wells from burn HTS DMVECs and 11 wells from NS DMVECs
				Transwell permeability assay	8 wells from burn HTS #1, 8 wells from NS
				PCR array	2 wells per burn HTS and NS yielded 2 arrays each
2	Full thickness excisional wounds (Day 0) (n = 4 HTS)		Day 70	Confirmatory qRT-PCR	3 wells per excisional HTS and NS
				ELISA	6 wells per excisional HTS and NS

The second type of injury was a full thickness excisional wounds without burn injury, as previously described (Animal 2, Fig. 1E)^32^. Two wounds were created on each flank of n = 2 pigs, resulting in 4 total wounds. Briefly, a dermatome set to 0.03″ was used for a total of 3 consecutive passes which resulted in injury down to the subcutaneous fat. The remaining pieces of viable dermis were excised with a goulian knife to achieve a plane of excision without any viable adnexal structures remaining. As described above, each week post wounding, the scars underwent cleansing and dressing changes. At Day 70 post-injury, skin and scars were excised and stored as described above until cell isolations occurred. The cells isolated from this injury type were used in confirmatory qRT-PCR and ELISA assays. 3 mm punch biopsies were also taken and fixed in formalin. The biopsies were subsequently paraffin embedded and stained with Verhoeff-Van Geison stain as previously described^33^.

### Cell isolation

Cells were isolated based on a modified method from Wang et al.^34^ Tissues were stored as described above for less than 4 h prior to processing. Excised scars and skin were sterilized through successive washes with 100% ethanol (X2), sterile water, and PBS (X2). The scar was then cut into thin strips (0.5 cm in width, unlimited length) and incubated with 1X dispase solution (Cellntec, Bern, Switzerland) overnight at 4 °C. The following day, the epidermis was peeled from the dermis and discarded. The dermis was then incubated in 1 mg/mL type I collagenase (MP Biomedical, Solon, Ohio) for 6 h at 37 °C After the incubation, PBS was added and 50 mL conical vials containing dermal samples were shaken vigorously to obtain single cells. The cell suspension was then filtered using a 70 um filter. Cells were pelleted by centrifugation and then reconstituted in DMVEC media (EGM-MV, Lonza, Rockville, MD) and seeded into T150 flasks as passage 1 (Corning, Lowell, MA). After the cells were allowed to adhere for at least 24 h, the flasks were rinsed and provided fresh media. The cells were then split 1:4 as passage 2.

### Magnetic sorting of DMVECs

After 3 days in culture, co-cultures of fibroblasts and DMVECs were clearly established based on cell morphology in the passage 2 cells. Cells were trypsinized and sorted using magnetic-activated cell sorting (MACS) with 5 ug/mL ulex europaeus agglutinin-1 lectin conjugated to biotin (B-1065, Vector Laboratories, Burlingame, CA). The manufacturer’s protocol for sorting with MS columns was followed and was performed as previously described (Milltenyi Biotec, Gaithersburg, MD)^35^. Anti-biotin beads were utilized (130-105-637, Milltenyi Biotec). Importantly, only 1e7 cells were sorted in each column to prevent the column from clogging. The cells that were retained on the column (DMVECs) were then seeded in T150 flasks as passage 3 cells. The fibroblasts that flowed through the column were seeded for morphological confirmation of fibroblast cells and then were discarded. There was very minimal fibroblast cell presence in these cultures, nevertheless, after these cultures reach confluency, they were sorted a final time using anti-fibroblast beads (130-0510601, Milltenyi Biotec) using MACS per the manufacturer’s protocol and sorted with MS columns. Cells were then reconstituted in DMVEC media and seeded into T150 flasks as passage 4 cells. Cells were then either used in chamberwells for immunocytochemistry, transwells for permeability assays, or were seeded into 6-well plates for the isolation of RNA for the PCR arrays.

### Chamberwell slides and phalloidin staining

Cells were seeded at 1e5 cells/cm^2^ in 8-well chamber-well slides (Millicell EZ slide, Millipore Sigma, Burlington, MA) that were pre-coated in rat tail coating solution at 5 μg/cm^2^ (Cell applications Inc., San Diego, CA). Cells were fixed with ice-cold 4% paraformaldehyde for 20 min at room temperature. Cells were then rinsed with PBS (X2). Cells were permeabilized with PBS containing 0.1% triton-X 100 for 5 min. The cells were then washed with PBS (X2). 300 μL of Texas red phalloidin (1 U/mL) and DAPI (1 μg/mL) diluted in PBS was incubated for 20 min at room temperature. The cells were then rinsed with PBS and water and mounted with fluoroshield. The stained cells were then imaged using a Zeiss Axio-cam with CY3 and DAPI fluorescent filters (Zeiss, city, state).

### Trans-endothelial electrical resistance and transwell permeability assay

Passage 4 DMVECs from Animal 1 (burn HTS and NS) were grown to confluency, trypsinized, and seeded at 50,000 cells/well onto 12-well transwell plates with 0.4 um pore polyester inserts that were tissue culture treated (CL53469, Millipore Sigma). The working volume for these plates was 0.5 mL in the apical well and 1.5 mL in the basal well. Each plate contained a blank well with no cells seeded in that well. Two different experiments were conducted. Experiment one assessed the growth rate of DMVECs derived from HTS or NS to form monolayers in transwell plates by assessing trans-endothelial electrical resistance (TEER)^36^. TEER was assessed at days 2, 3, and 4 after seeding. Each plate was given fresh media and allowed to equilibrate to room temperature for 30 min. A sterilized probe was then calibrated to room temperature (Millicell ERS-2 Voltohmmeter, Millipore-Sigma, Burlington, MA). Each well was assessed in triplicate. Prior literature showed that TEER ≥ 30 Ohms indicated the formation of a monolayer of cells. Therefore, at day 4, when all wells reached ≥ 30 Ohms, a transwell permeability assay was conducted. On day 4 post-seeding, the media was aspirated from both wells. Serum-free media was added to the basal well and 250 ug/mL FITC-dextran (40Kda, Millipore-Sigma) in PBS was added to the apical well and dwelled for 2 h. The apical well was removed and the FITC-dextran that was diffused into the bottom well was measured by spectrophotometry (Ex: 485 nm and Em: 528 nm (FilterMax F5, Molecular Devices, San Jose, CA)) in 96-well plates. From each basal well, n = 3 technical replicates were plated in the 96-well plates along with a 5-point standard curve that ranged from 0 to 48 μg/mL of 40-KDa FITC-dextran. For the comparison of burn HTS versus NS DMVECs, the data is expressed as concentration. These cells did not undergo in vitro cellular injury, they were simply isolated from burn HTSs or NS.

In the second experiment, burn HTS and NS DMVECs were seeded on transwell inserts and grown to confluence confirmed by TEER ≥ 30 Ohms. Permeability assays were performed as described above. Controls received no treatment. A sub-group of wells were injured. Monolayer cellular damage in vitro was induced with exposure to a combined treatment with 1 uM epinephrine and 1 mM H_2_O_2_ for 2 h as previously described. For the comparison of the effect of injuries to each cell types, the data is expressed as a permeability index developed by Maruo et al. (1992) which corrects for tracer diffusion through blank inserts and normalizes diffusion to control monolayers^37^. In calculating permeability index, for experiment 1, the control was NS DMVECs and the experimental condition was burn HTS DMVECs. For experiment 2, control was the un-injured burn HTS or NS DMVECs. The experimental condition was the epinephrine/ H_2_O_2_ cellular injury that occurred in vitro for each cell type (burn HTS and NS DMVECs).

### Cell culture and RNA isolation

Passage 4 DMVECs isolated as described above from Animal 1 (burn HTS (n = 2) and NS (n = 1)) DMVECs were seeded at 100,000 cells/cm^2^ in 6-well plates (n = 6 replicates per scar/skin sample). The following day, images were acquired with phase-contrast microscopy to visualize cell morphology. Then, cells were lysed with Trizol tissue reagent and stored at -80 °C until isolation of RNA. Cell homogenate treated with 1 mL of Trizol were incubated for 5 min prior to the addition of 0.2 mL of chloroform per 1 mL of Trizol used, mixtures were vortexed, and sat at room temperature for 3 min. Mixtures were centrifuged at 12,000xg for 15 min at 4 °C and aqueous phases were transferred to RNase-free microcentrifuge tubes to which 1.5 volumes of 100% molecular grade ethanol was slowly added. New mixtures were vortexed and the samples were loaded into a Qiagen RNeasy spin column. The samples were then washed in successive rounds of Buffer RW1 and Buffer RPE, followed by sample elution in RNase-free water. RNA was quantified using the Nanodrop 2000. RNA was stored at − 80 °C until use.

### PCR array analysis

DMVECs isolated from Animal 1 burn HTS (n = 2) and NS (n = 1) were used to run 84-gene angiogenesis-specific PCR arrays (Qiagen, Valencia, CA). 300 ng of RNA was used to create cDNA using the RT2 first strand kit according to the manufacturer’s instructions (Qiagen) with a cutoff value of Ct = 35. Samples were normalized to housekeeping genes glyceraldehyde 3 phosphate (GAPDH) and ribosomal protein ligand 13a (RPL13a). For each scar and skin area, RNA from two different cell culture wells was run on a separate array for a total of 6 arrays. The scars were normalized to the average of the normal skin samples. Fold change > or < 2 was set as the threshold for significance. Hierarchical clustering and heat map analysis were performed using the GeneGlobe Data Analysis Center (Qiagen).

### Confirmatory quantitative real-time polymerase chain reaction (qRT-PCR)

DMVECs were isolated as described above from Animal 2 from excisional HTS (n = 4) and NS (n = 1). DMVECs were seeded in 6-well plates (n = 6 wells per scar or skin area) at 20,000 cells/well and were grown for 5 days with media changes every other day. At day 5, the conditioned-media was collected and saved at − 80 °C. The cells were then lysed in Trizol reagent as above and RNA was isolated as described. Mastermix was prepped using the ratio of 12.5 μL of iScript Universal SYBR Green (172-5151, Bio-Rad, Hercules California), 0.75 μL of 10 uM forward and 0.75 μL of 10 uM reverse primers (Integrated DNA technologies Coralville, IA or Qiagen), 0.5 μL of RNase free water, and 0.5 μL of reverse transcriptase per reaction. No Reverse Transcriptase (NRT) mix was made using the same contents and ratios as the Mastermix except for the reverse transcriptase. 15 μL of mastermix was transferred per well into 96-well plate (Bio-Rad). Duplicates of thawed 1 ng/μL RNA samples were added in 10 μL quantities per well aside from the not template controls (NTC), which were filled with 10 μL of RNase free water. The plate was centrifuged at 1000 rpm for 3 min and ran at annealing temperatures appropriate for the primers used (Table 2). GAPDH was used as the housekeeping gene^38^ and excisional HTS was normalized to NS to obtain fold change.

**Table 2:** Primer sequences for genes used in confirmatory qRT-PCR.

Gene and ID	Forward primer	Reverse primer	Annealing temperature
IL6	5′-CCC TGA GGC AAA AGG GAA AGA-3′	5′-CTC AGG TGC CCC AGC TAC AT-3′	54
ANG	5′-AAT GAA GCA ACG AGG CCT GA-3′	5′-GGT TGG ACC CTC CCT TAT GC-3′	54
ANGPT1	5′-TCC ACG CTG AAC GGT TAC AC-3′	5′-TTT CCC TCT CAA AGA ACG CC-3′	53
ET-1	Proprietary		55
TGFβ3	Proprietary		55

### ELISA

The conditioned media from the DMVECs isolated from Animal 2 as described above was used in ELISA assays to assess the levels of secreted cytokines. Prior to assay, the 2 mL of conditioned media per well was concentrated to ~ 500 μL using an Amicon ultra-0.5 centrifugal filter unit according to the manufacturer’s protocol (3KDa, Millipore Sigma). Concentrated protein was diluted 1:10 and used in ELISAs for transforming growth factor beta 3 (TGFβ3) (MBS2703941, MyBiosource, San Diego, CA), interleukin-6 (IL6) (P26893, RayBiotech, Peachtree Corners, GA), angiopoietin-1 (ANGPT1) (Q9BDY8, RayBiotech), angiogenin (ANG) (MBS456579, MyBiosource), and endothelin-1 (ET-1) (MBS262991, MyBiosource) according to the manufacturer’s instructions. After bringing kit components and samples to room temperature, standard was reconstituted to produce a stock solution. Wells of the pre-coated 96-well plate were designated and filled with the 7 standards, 1 blank, and samples before being covered with a plate sealer and incubated at 37 °C. The liquid contents were then removed without washing any of the wells. Detection reagent A was added in quantities of 100 μL to each well after which the plate was sealed and incubated for an hour at the same temperature. The contents were aspirated and the wells washed 3 times. Each well was filled with 100 μL of detection reagent B, covered with the plate sealer, and incubated at 37 °C for the appropriate time. The wash steps were repeated for a total of 5 washes and 90 uL of substrate solution was added to each well. The plate was covered with a new plate sealer and incubated at 37 °C for the appropriate time while protected from light before 50 uL of stop solution was pipetted into each well. The side of the plate was gently mixed to cause a uniform color change. The plate was then read via microplate reader at 450 nm.

### Statistical analysis

To compare melanin indices between hyper-, hypo-, and normally pigmented areas, one-way ANOVA was used with Tukey’s correction for multiple comparisons. Elasticity and trans-epidermal water loss (TEWL) between HTS and NS were analyzed with an un-paired t-test Mann–Whitney test. Student’s t-tests were used to evaluate changes between NS and HTS gene and protein expression and *p* < 0.05 was considered significant. Unpaired t-tests were used to evaluate transwell concentrations between uninjured burn HTS DMVECs vs. NS DMVECs. Transwell concentrations between burn HTS ± injury and NS ± injury was analyzed using one-way ANOVA with Tukey’s correction for multiple comparisons. Protein expression measured by ELISA for excisional HTS 1, HTS 2, HTS, HTS 3, HTS 4, and NS, was analyzed using one-way ANOVA with Dunnet’s correction for multiple comparisons.

## Results

### Phenotypic characterization of hypertrophic scars

Burn HTS at Day 86 resulting from full thickness burn injuries followed by excision contain many of the known features of human HTS (Fig. 1A). They are dyschromic with hyper- and hypo-pigmentation compared to uninjured skin (Hyper = 845 ± 21.67 vs. Hypo = 652.5 ± 30.17 vs. NS = 740.7 ± 4.67, n = 2 scars/skin areas) (Fig. 1C). They are also stiff/non-elastic (HTS = 116.6 ± 14.25 vs. NS = 49.25 ± 6.58 N/m, n = 2 scars/skin areas) (Fig. 1D). Last, they have increased TEWL compared to un-injured skin (HTS = 16.88 ± 4.26 vs. NS = 11.45 ± 0.22 g/m^2^h) (Fig. 1E). Excisional HTS at Day 70 resulting from full thickness excisional wounds is very similar in phenotype to the burn HTSs studied (Fig. 1B). They are dyschromic with hyper- and hypo-pigmentation compared to uninjured skin (Hyper = 882.3 ± 6.15 vs. Hypo = 621.2 ± 5.35 vs. NS = 798.2 ± 9.11, n = 4 scars/skin areas, *p* < 0.0001) (Fig. 1C). They are also stiff/non-elastic (HTS = 243.6 ± 1.97 vs. NS = 78.17 ± 12.81 N/m, n = 4 scars/skin areas, *p* < 0.05), however to a greater degree than the burn HTS (Excisional HTS = 243.6 ± 1.97 vs. Burn HTS = 116.6 ± 14.25) (Fig. 1D). They trended towards increased TEWL compared to un-injured skin (HTS = 15.78 ± 4.78 vs. NS = 7.32 ± 1.54 g/m^2^h) (Fig. 1E). We have previously reported the presence of hypervascularity in HTS with the use of alpha-smooth muscle actin staining^29^. HTSs from the current study appear to be hypervascular compared to normal skin based on representative sections stained using Verhoeff-Van Gieson stain to observe blood vessels (Fig. 1F).

### DMVEC cell morphology, RNA content, and phalloidin staining

**Figure 2:**
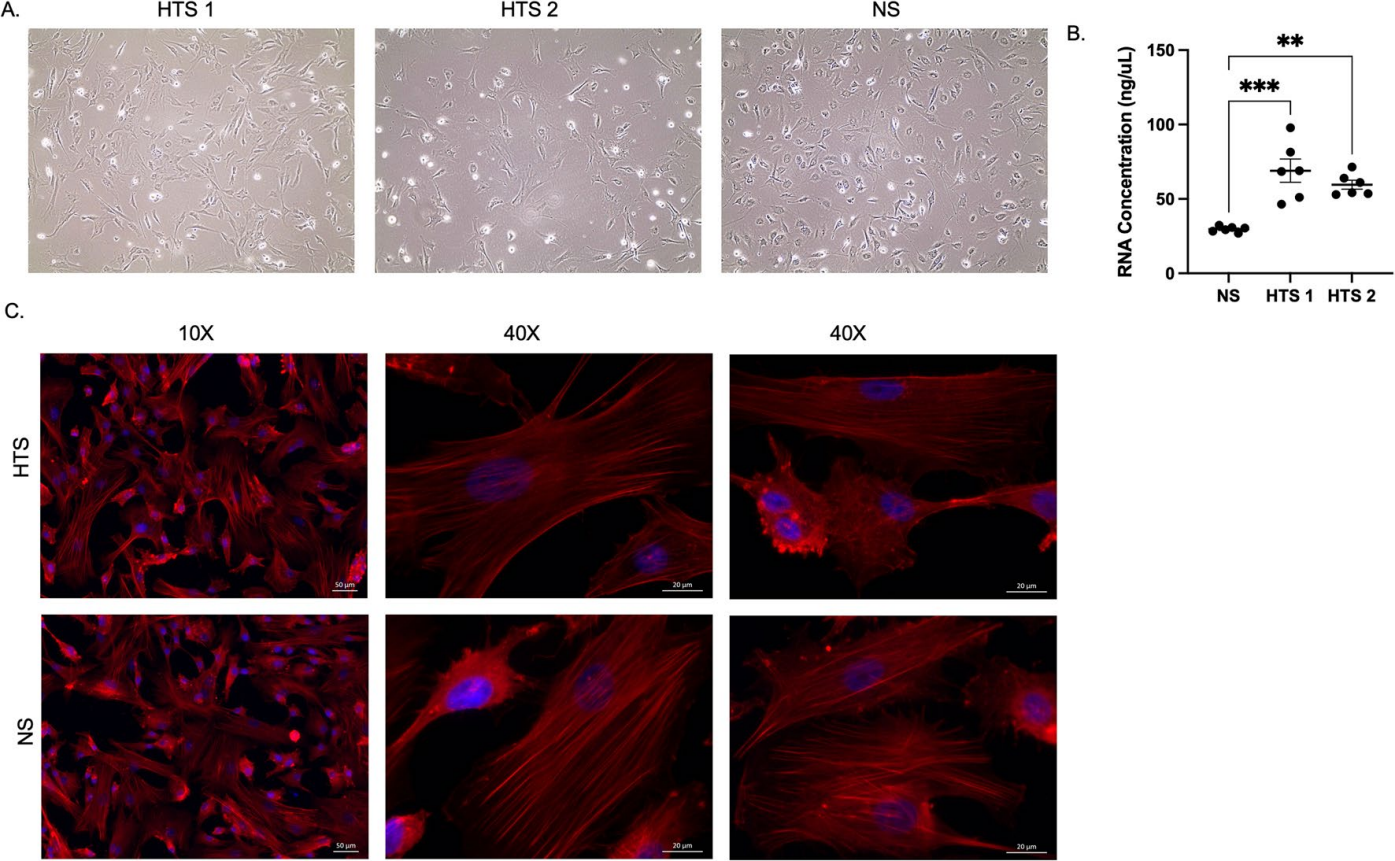
DMVECs from HTS and NS exhibit different morphology in vitro and yield different RNA amounts. Phase contrast imaging of HTS and NS DMVECs (**A**) and RNA concentration from these cells (**B**). Texas-red phalloid (red) and DAPI (blue) staining of HTS and NS DMVECs (**C**). *p* < 0.05*, *p* < 0.01**, *p* < 0.001***.

HTS DMVECs were larger in size compared to NS DMVECs (Fig. 2A). HTS DMVECs, despite seeding the same number of cells per well, resulted in significantly higher RNA content compared to NS (*p* < 0.01) (Fig. 2B). HTS and NS DMVECs stained for phalloidin can be visualized in Fig. 2C*.*

### TEER and transwell permeability assays

**Figure 3:**
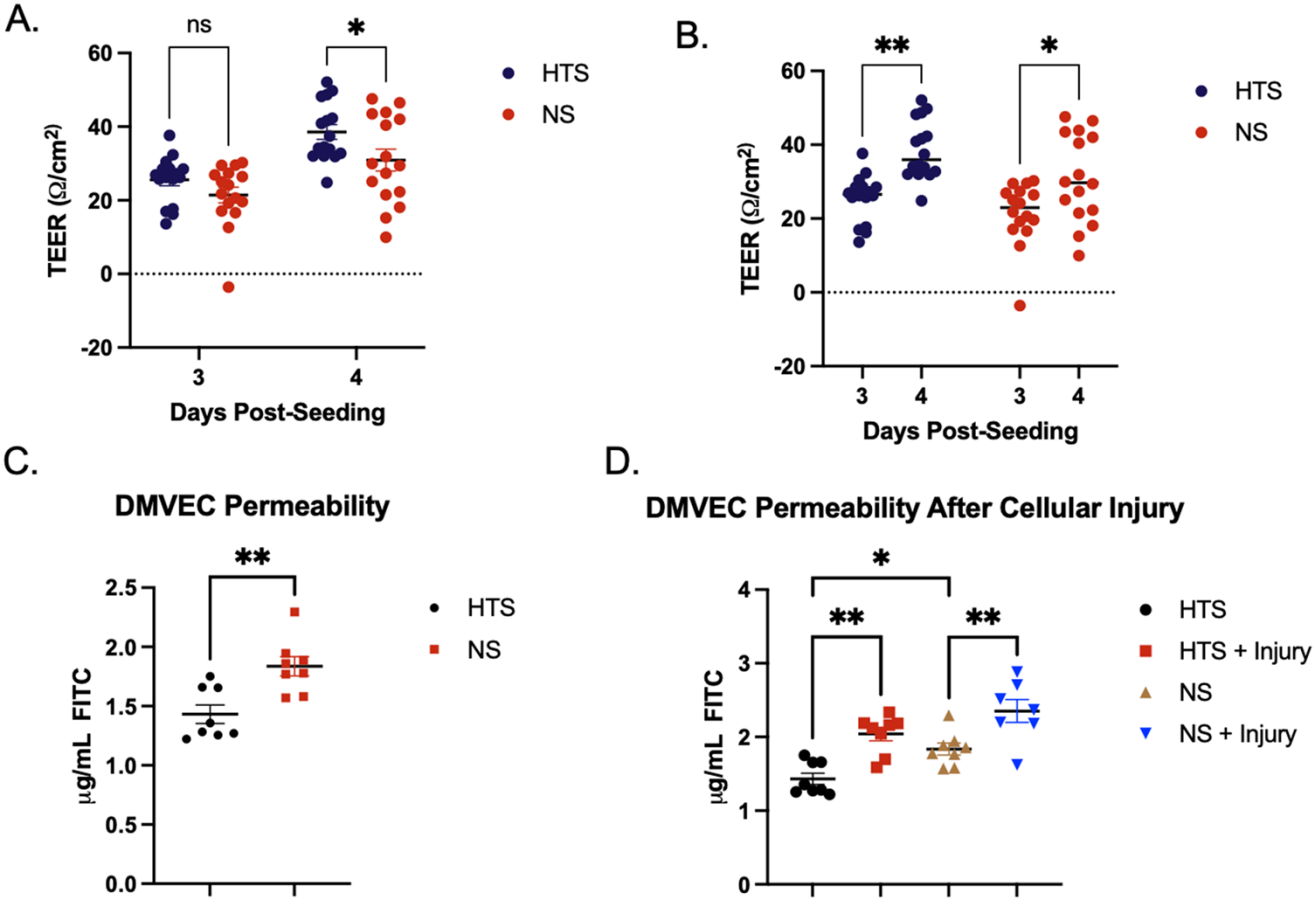
DMVECs from burn HTS have decreased barrier function and increased permeability compared to NS DMVECs. TEER was measured after 3 and 4 days in culture from burn HTS and NS DMVECs. Comparing burn HTS versus NS (**A**) and burn HTS and NS over time (**B**). FITC-dextran assays were used to assess permeability between burn HTS and NS (**C**) and in cells after injury (**D**). *p* < 0.05*, *p* < 0.01**.

TEER was increased in burn HTS DMVECs compared to NS DMVECs at days 3 and 4 (day 3 = 25.55 ± 1.60 vs. 21.44.93 ± 2.11 Ω/cm^2^, day 4 = 38.57 ± 1.98 vs. 30.92 ± 2.97 Ω/cm^2^ (n = 16, *p* < 0.05) (Fig. 3A). In addition, TEER increased significantly each day in culture in both burn HTS and NS DMVECs (n = 16, *p* < 0.05) (Fig. 3B). An increase in TEER indicates that tighter endothelial barriers were formed suggesting that burn HTS would allow decreased permeability of a tracer molecule compared to NS DMVECs.

On day 4, when all wells reached TEER ≥ 30 Ohms, FITC-dextran permeability was increased in NS DMVECs compared to burn HTS DMVECs without any cellular injury in vitro (1.83 ± 0.08 vs. 1.43 ± 0.08 μg/mL, n = 8, *p* = 0.0028) (Fig. 3C). A permeability index, which incorporates the endogenous diffusion of the blank well in each plate, as well as the control for each experiment (in this case NS DMVECs), allowed for comparison of permeability across experimental conditions. Under normal culturing conditions without any cellular injury in vitro, burn HTS DMVECs were 17.69% less permeable than NS DMVECs (*p* = 0.0002), data in agreement with Fig. 1C that showed greater FIT-C diffusion across the cellular layer.

Injury with combined treatment of epinephrine and hydrogen peroxide induced statistically significant increases in permeability in both burn HTS and NS DMVECs (burn HTS = 1.43 ± 0.08 vs. burn HTS with injury = 2.04 ± 0.09 μg/mL, n = 8, *p* = 0.0011) (NS = 1.83 ± 0.08 vs. NS with injury = 2.35 ± 0.16 μg/mL, n = 8, *p* = 0.0082) (Fig. 3D). After epinphrine/H_2_O_2_ injury, NS DMVECs were 28.4% more permeable, while burn HTS DMVECs were only 18.8% more permeable than uninjured controls (28.4 ± 4.8 vs 18.8 ± 2.8; *p* = 0.11). This indicates that burn HTS DMVECs had a larger response to in vitro cellular injury compared to the NS DMVECs.

### PCR array and hierarchical clustering

**Figure 4:**
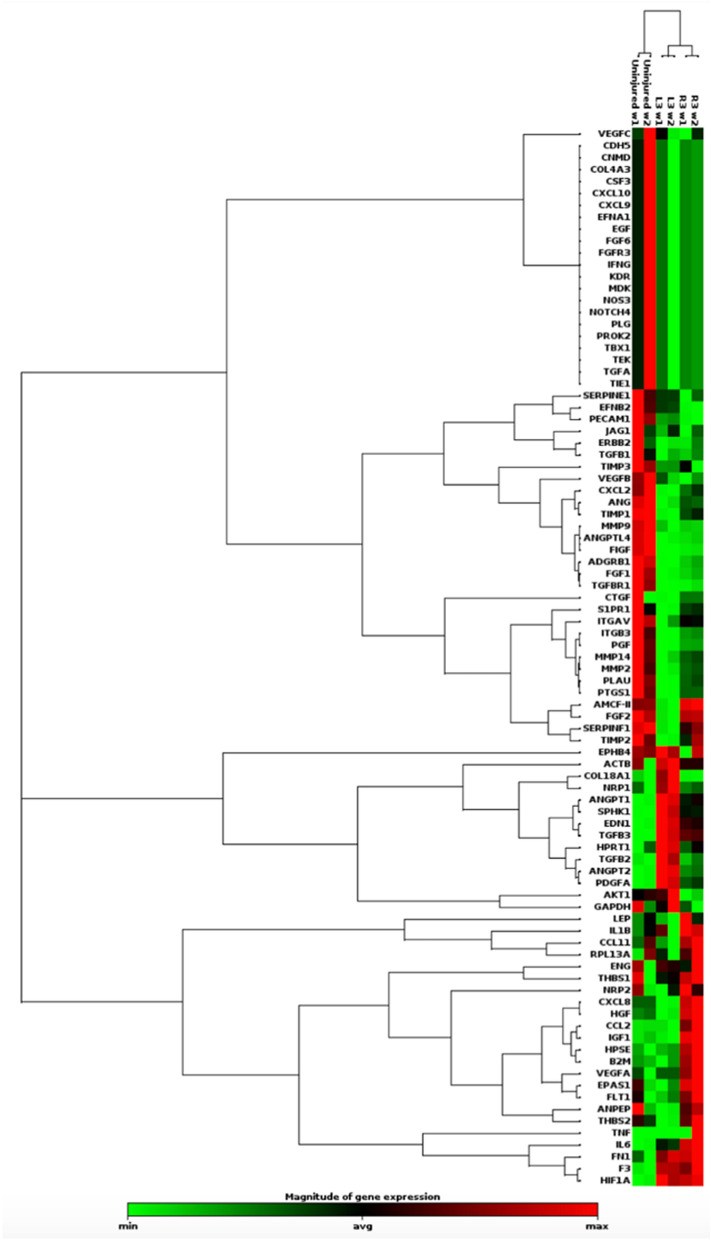
Gene expression in DMVECs differ between burn HTS compared to NS and vary amongst individual HTS. Hierarchical clustergram visually presents gene expression from the PCR array in a heat map format for 2 different scars from animal 1 compared to uninjured skin. Green = upregulation, red = downregulation, black = no detectable change.

Isolated DMVEC RNA was analyzed with an angiogenesis-specific 84 gene PCR array which then underwent hierarchical clustering. Hierarchical clustergram (Fig. 4) allowed for visualization of gene expression in a heat map format for the two burn HTS compared to uninjured skin. Samples clustered DMVECs from HTS vs NS at the highest level. They also clustered by scar at a secondary level. Therefore, while there was variability in the mRNA expression profile of each scar, the profile of scar DMVECs overall was substantially different compared to that of NS DMVECs.

**Figure 5:**
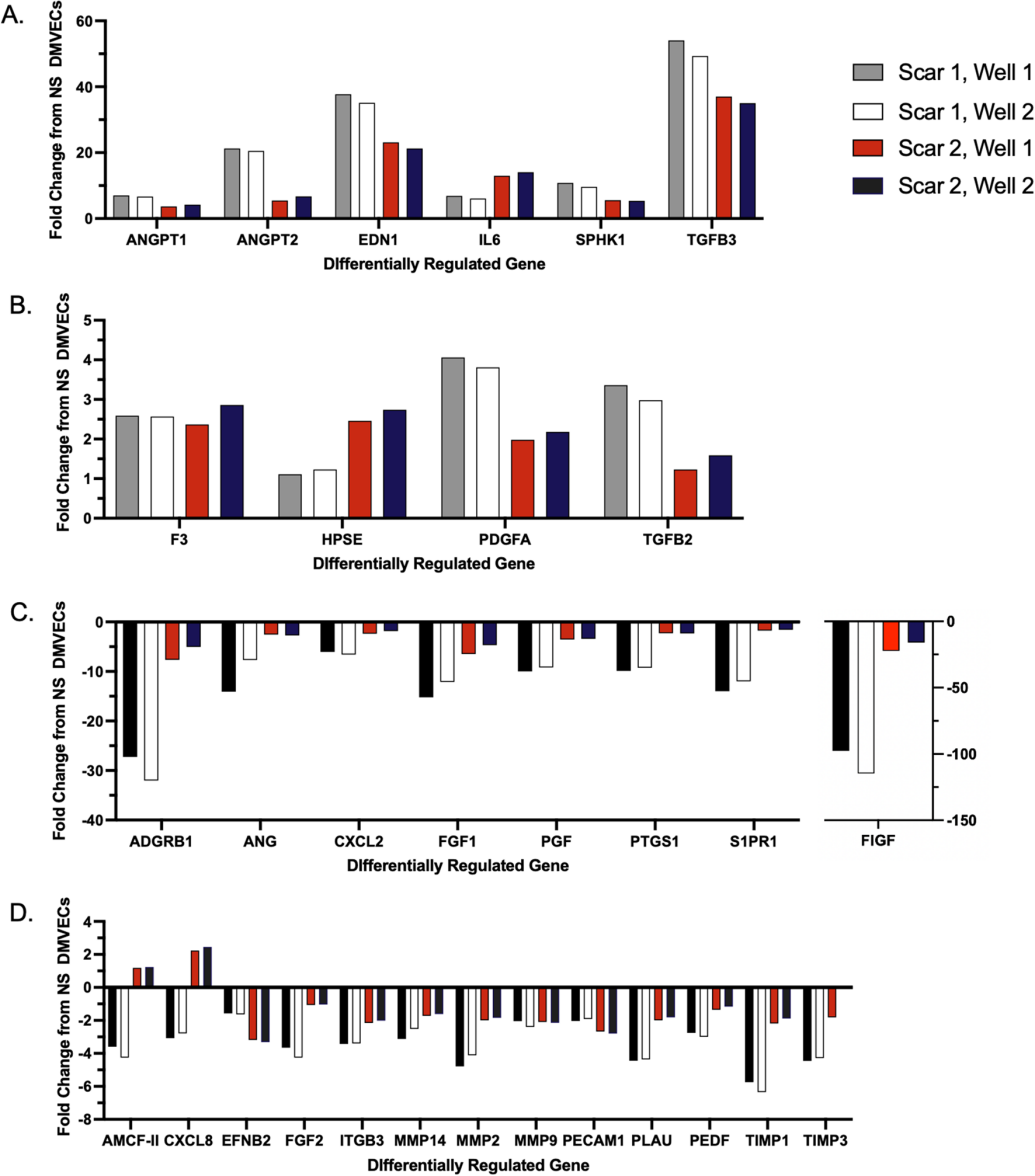
DMVECs from burn HTS differentially express 31 out of 84 genes relative to NS DMVECs. PCR array measured mRNA expression between burn HTS and NS DMVECs. Gene expression upregulated greater than 5-fold in HTS versus NS (**A**), gene expression upregulated less than 5-fold in burn HTS versus NS (**B**), gene expression downregulated greater than -5-fold in burn HTS versus NS (**C**), and gene expression downregulated less than -5-fold in HTS versus NS (**D**).

Based on PCR array, 31 genes were differentially expressed between scar and skin DMVECs (> or < 2-fold change). Of those 31 genes, 10 genes were upregulated with 6 of these being greater than 5-fold (Fig. 5A) and 4 of these > 2 < 5-fold (Fig. 5B). 21 genes were down regulated with 8 of these being less than 5-fold (Fig. 5C) and 13 < -2 > -5-fold (Fig. 5D). Genes of interest were selected to undergo confirmation and analysis with qRT-PCR and ELISA in different scars with increased n values based on the degree of up- or down-regulation.

### Confirmatory qRT-PCR

qRT-PCR analysis was used for confirmation of gene expression that was measured by PCR array. HTS and NS samples for qRT-PCR were obtained from a different pig (Animal 2, excisional HTS) than what was used for PCR array.

**Figure 6:**
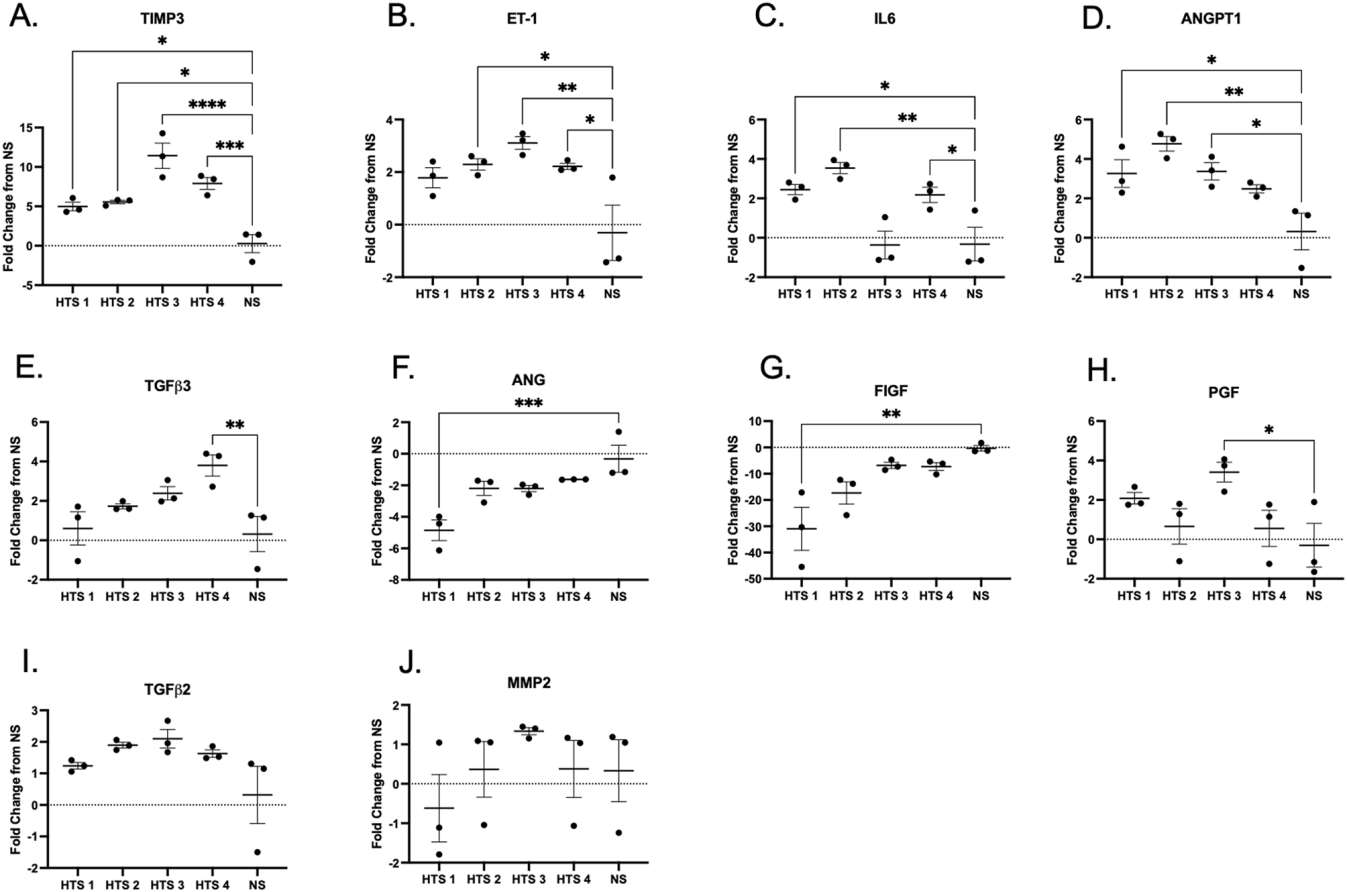
Differences in expression of highly differentiated genes are confirmed with qRT-PCR. Animal 2 excisional HTS DMVECs and NS DMVECs gene expression measured by qRT-PCR for TIMP-3 (**A**), ET-1 (**B**), IL6 (**C**), ANGPT1 (**D**), TGFβ3 (**E**), ANG (**F**), FIGF (**G**), PGF (**H**), TFGβ2 (**I**), and MMP2 (**J**). *p* < 0.05*, *p* < 0.01**, *p* < 0.001***, *p* < 0.0001****.

There were 4 genes (tissue inhibitor of matrix metalloproteinase-3 (TIMP3), ET-1, IL6, and ANGPT1) that were significantly differentially regulated in more than one scar compared to NS (Fig. 6A–D). The greatest fold change for TIMP3 was 11.43-fold in HTS 3, with an average of 7.47-fold and 4/4 scars were significantly different from NS (Fig. 6A, *p* < 0.05). The highest fold increase of ET-1 in HTS DMVECs was 3.11 in HTS 3, with an average of 2.35-fold and ¾ scars were significantly different from NS (Fig. 6B, *p* < 0.05). The highest fold increase of IL-6 was 3.54-fold in HTS 2 DMVECs, with an average of 1.95-fold and ¾ scars were significantly different from NS (Fig. 6C, *p* < 0.05). The highest fold increase for ANGPT1 was 4.77 in HTS 2, with an average of 3.47-fold and ¾ scars were significantly different from NS (Fig. 6D, *p* < 0.05).

There were 4 genes (TGFβ3, ANG, c-Fos-Induced Growth Factor (FIGF), and placental growth factor (PGF)) that were only significantly differentially regulated in one out of 4 scars (Fig. 6E–H). A 3.80-fold increase of TGFβ3 mRNA expression was the highest fold change found for HTS 4 DMVECs, with an average of 2.13-fold among scar samples, and only 1/4 scars were significantly different from NS (Fig. 6E, *p* < 0.001). The greatest fold decrease for ANG was a -4.85 in HTS 1 DMVECs (Fig. 6F, *p* < 0.001), with an average of -2.72-fold and only 1/4 scars were significantly different from NS. The greatest fold decrease of FIGF was -30.96-fold in HTS 1 (Fig. 6G, *p* < 0.01), with an average of -15.59-fold and only 1/4 scars were significantly different from NS. The greatest fold change for PGF was 3.41-fold in HTS 3, with an average of 1.67-fold and only 1/4 scars were significantly different from NS (Fig. 6H, *p* < 0.05). Neither transforming growth factor beta 2 (TGFβ2) nor matrix metalloproteinase-2 (MMP2) showed significant fold changes compared to NS (Fig. 6I,J).

### ELISA

**Figure 7:**
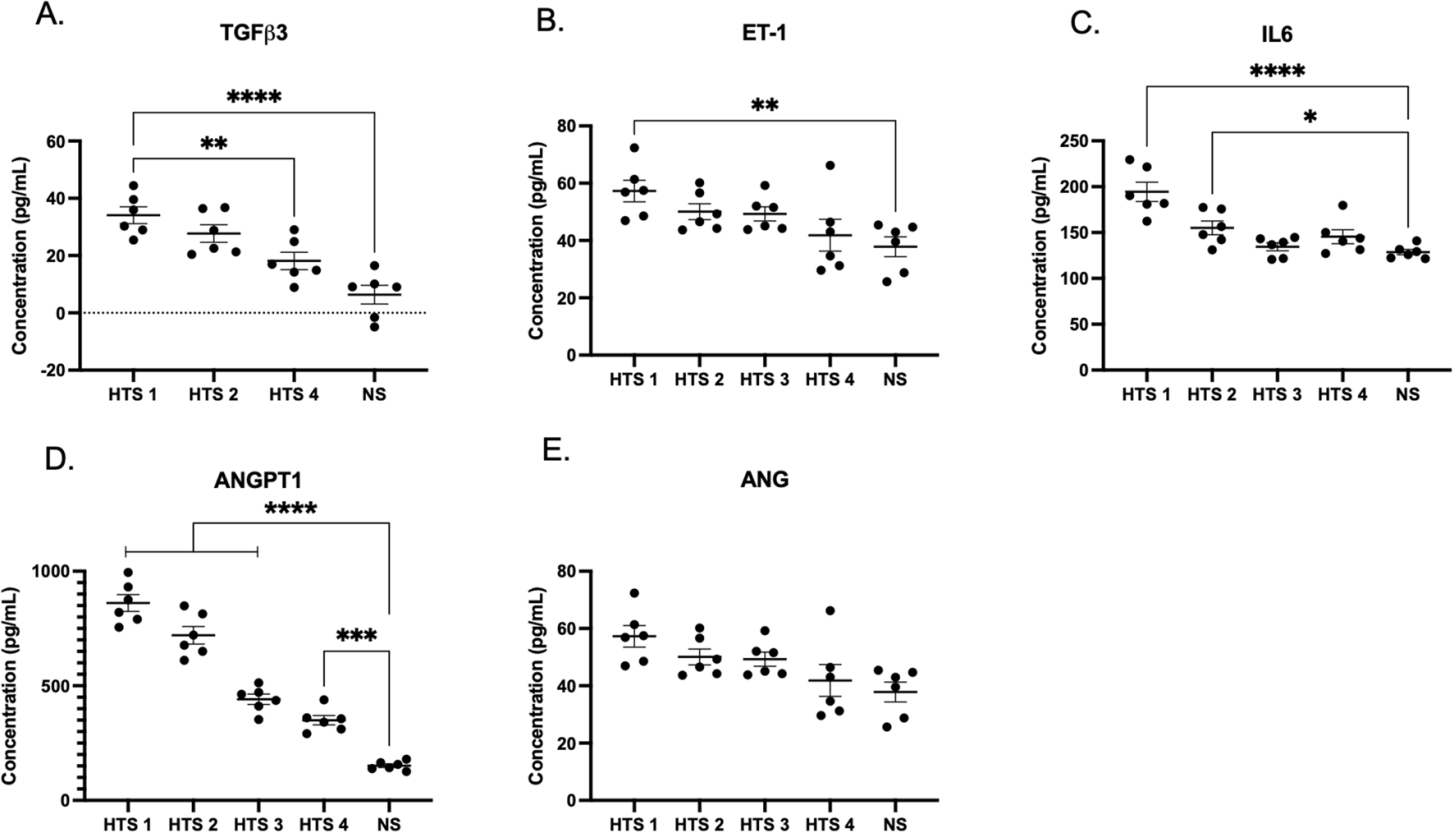
DMVECs from HTS differentially regulate protein expression compared to NS DMVECs in accordance with differentially regulated gene expression. ELISA measured expression of select proteins from excisional HTS DMVECs and NS DMVECs. Expression of TGFβ3 (**A**), ET-1 (**B**), IL6 (**C**), ANGPT1 (**D**), and ANG (**E**). *p* < 0.05*, *p* < 0.01**, *p* < 0.001***, *p* < 0.0001****.

Conditioned media in which the DMVECs grew was analyzed using ELISA for confirmation of protein secretion. The scar and skin samples that underwent confirmatory qRT-PCR were used for analysis with ELISA. TGFβ3 protein secretion for scars 1, 2, and 4, was measured as 34.11 ± 7.15, 27.72 ± 7.49, and 18.15 ± 7.43 pg/mL respectively, which were significantly increased compared to NS at 6.38 ± 8.02 pg/mL. Relative to normal skin, scars had increased fold changes of 5.35, 4.35, and 2.85, respectively (Fig. 7A).

ET-1 protein secretion for scars 1, 2, 3, and 4 was 57.27 ± 9.24, 50.08 ± 6.81, 49.32 ± 6.07, and 41.87 ± 13.64 pg/mL respectively, compared to NS at 37.85 ± 8.54 pg/mL. Relative to NS, the highest fold change was a 1.51-fold increase (Fig. 7B).

IL-6 protein secretion for scars 1, 2, 3, and 4 was 194.41 ± 25.88, 155.10 ± 18.52, 132.68 ± 10.77, and 145.44 ± 18.69 pg/mL respectively compared to NS at 128.63 ± 7.16 pg/mL. Relative to NS, the highest fold change was a 1.51-fold increase (Fig. 7C).

ANGPT1 protein secretion for scars 1, 2, 3, and 4 was 861.24 ± 90.10, 720.44 ± 93.8, 441.11 ± 55.17, and 349.99 ± NS skin at 151.50 ± 19.49 pg/mL. Relative to NS, scars had increased fold changes of 5.68, 4.76, 2.91, and 2.31, respectively (Fig. 7D).

ANG protein secretion for scars 1, 2, 3, and 4 was 56.26 ± 9.02, 64.33 ± 8.92, 73.16 ± 6.14, 60.15 ± 17.02 pg/mL respectively compared to NS at 68.0 ± 8.2 pg/mL. Relative to NS, scars had fold changes that ranged from 0.82–1.07-fold; therefore, scar secretion of ANG was similar or decreased compared to NS (Fig. 7E). Data supporting the results is available in the Supplementary File.

## Discussion

Interactions between endothelial cells, the ECM, and other cellular components such as fibroblasts and inflammatory cells play an important role in the process of angiogenesis and wound healing^39–41^. During wound healing there is creation of a dense but poorly organized capillary bed that is eventually remodeled to normal density and capillary structure^42^. Persistent inflammation and excessive angiogenesis is thought to contribute to the formation of scars. This can be contrasted to fetal skin and oral mucosa which exhibit scarless or near scarless wound repair while involving reduced inflammation and capillary growth^43^. The integrity of the endothelium appears to be disrupted in the setting of burn injury, leading to a state of endotheliopathy^9^. Abnormal paracrine and autocrine signaling and interactions involving endothelial cells is postulated to contribute to the formation of subsequent HTS.

This study provides a broad functional analysis of DMVECs from hypertrophic burn scars. DMVECs derived from HTS were larger than NS DMVECs in culture and produced more total RNA per cell. In accordance with what has been reported in the literature in vivo*,* HTS DMVECs formed cell monolayers that had increased overall barrier function compared to NS DMVECs by TEER and decreased permeability by FITC-dextran assays. HTS DMVECs were ~ 18% less permeable than NS DMVECs by permeability index. This data is in line with in vivo reports of blood vessel occlusion in HTS. In addition, HTS DMVECs responded differently to injury compared to NS DMVECs. While both cell types showed a statistically significant increase in permeability after injury, the response in HTS was only 18% while the NS response was 28% more permeable. This data suggests that HTS DMVECs may respond differently to changes in the scar environment such as reactive oxygen species levels (such as H_2_O_2_). The alteration of HTS DMVEC occlusion and response to injury may be key in elucidating treatments for scars. By outlining the mechanism by which HTS DMVECs contribute to blood vessel occlusion, this process may be prevented. With fewer occluded blood vessels, it is possible that far fewer blood vessels will grow through angiogenesis, and symptoms such as erythema will be reduced.

Transcript levels and protein expression in DMVECs from HTS were found to differ compared to DMVECs from normal skin. PCR array allowed for a shotgun approach to generate an overall profile of gene expression in DMVECs. The array was specific for genes known to be involved in the process of angiogenesis, which is typically thought to be increased in early HTS and decreased as the scar matures^21,43,44^. Of the 84 genes that were assayed, 31 genes were differentially expressed between skin and scar DMVECs. Previously, Matsumoto et al*.* conducted an analysis of DMVECs in keloids with the use of microarray and found a difference of 15 upregulated genes and 3 downregulated genes in keloid DMVECs compared to normal skin^45^. The study emphasized the upregulation of serine peptidase inhibitor class A member 3 (SERPINA3) and Laminin Subunit Gamma 2 (LAMC2), neither of which were included in our microarray except for SERPINF1, which is in the serpin family and was downregulated in HTS DMVECs. SERPINF1 is known to inhibit angiogenesis, therefore its downregulation points to a pro-angiogenic environment.

TGFβ3 was upregulated in scars compared to normal skin at the gene and protein levels which confirmed our finding from the PCR array. Transforming growth factor family has been extensively studied in hypertrophic scar. It is primarily associated with stimulating collagen and ECM deposition from fibroblasts leading to tissue fibrosis. Typically transforming growth factor 1 (TGFβ1) and TGFβ2 are recognized as causing excess collagen deposition^46^. In contrast TGFβ3 is recognized as reducing connective tissue deposition, predominantly in the later stages of wound healing^47^. Based on qT-PCR results, TGFβ2 gene expression was also increased in HTS DMVEC compared to normal skin. If TGFβ2 protein secretion is confirmed to be in accordance with its gene expression, it would appear that DMVECs contribute to both pro- and anti-fibrotic signaling.

Previously, Peltonen et al. demonstrated localization of TGFβ1 to neovascular endothelial cells in keloids, suggesting that the endothelial cells could activate adjacent fibroblasts to initiate fibrotic reactions by secreting TGFβ^48^. TGFβ can induce the process of EndoMT which further contributes to the development of fibrosis^49^. Based on our findings, autocrine signaling of TGFβ from endothelial cells is a potential mechanism contributing to HTS development. A potential role of endothelial cells (ECs) was described by Monsuur et al. in which ECs were found to stimulate adipose tissue-derived mesenchymal stromal cells (ASC) leading to contraction of the dermal matrix via reduction of follistatin^50^. This contraction was in part mediated via the TGFβ pathway. Prior to our work, TGFβ3 was thought to be secreted predominantly from myofibroblasts within the dermis. Our analysis is in accordance with a 2021 study^51^ of keloid-derived endothelial cells where the TGFβ super family was identified as a main pathway in these cells compared to NS.

Similar to TGFβ3, ET-1 was confirmed to be upregulated in scar vs. skin DMVECs by gene and protein level analysis which confirmed the array**.** ET-1 is known as a potent vasoconstrictor polypeptide mainly produced by vascular endothelial cells and its role in tissue fibrosis has been studied in multiple different organ systems including, renal, cardiovascular, and pulmonary systems^52^. A study in 2016 on ET-1 from DMVECS showed that ET-1 induced myofibroblast differentiation and collagen synthesis in cultured human dermal fibroblasts^53^. ET-1 can also promote myofibroblast resistance to apoptosis through induction of Survivin expression^54^. Lagares et al*.* demonstrated that TGFβ1 induced ET-1 expression in human dermal fibroblasts, and overexpression of these two factors was associated with accelerated wound closure, increased fibrogenesis, and excessive scarring^55^. Treatment with bosentan, an ET-1 receptor antagonist, prevented the fibrotic response to TGFβ in their mouse model of bleomycin-skin fibrosis. Profibrotic effects of ET-1 have been demonstrated through potentiation of TGFβ1-induced EndoMT^56^.

Our findings suggest that ET-1 secretion from DMVECs plays a critical role in the known ECM deposition process by contributing to paracrine signaling with fibroblasts. In contrast, Xi-Qiao et al*.* demonstrated that DMVECs from HTS secreted less ET-1, as well as less TGFβ1, VEGF, platelet derived growth factor (PDGF), and basic fibroblast growth factor (bFGF), compared to NS DMVECs^57^. More recently, Xi-Qiao et al*.* in 2017 evaluated ECs and fibroblasts in HTS classified as proliferative, regressive, or mature^58^. ECs secreted significantly less TGFβ1, VEGF, PDGF, ET-1, and bFGF in proliferative and regressive scar compared to normal skin. Furthermore, EC medium from regressive scar, which were defined as being at least 2 years old, inhibited fibroblast viability and collagen production and induced apoptosis. Based on our PCR array results, PDGFA and ET-1 gene expression was upregulated while fibroblast growth factor 2 (FGF2) (gene for bFGF), and FIGF (gene for VEGF-D) gene expression was downregulated in DMVECs from HTS. PDGFA protein and ET-1 from DMVECs both can contribute to the process of fibrosis^59^. The HTSs in this study were of similar age as scars classified as proliferative scars. The group found the microvasculature to be surrounded by large collagen deposits, and postulates that this causes mechanical constriction leading to occlusion and cellular damage.

IL6 was likewise up regulated in scars vs. skin. The cytokine IL6 is primarily known for its role in inducing inflammation and can be present at sites of both acute and chronic inflammation. In a IL6 deficient mouse model, it has been reported that IL6 has the ability to induce the expression of TGFβ1 from dermal fibroblasts^60^. The administration of IL6 to fetal wounds, which heal with minimal inflammation, results in scar formation^61^. Our findings show that endothelial cells produce high levels of cytokines which may affect fibroblast and keratinocyte cell signaling as well as contribute to prolonged inflammation. IL6 has been shown to be involved in EndoMT in autophagy-deficiency-induced mice, leading to tissue fibrosis^62^. Culture medium of autophagy-deficient human DMVECs contained increased IL6 and EndoMT was inhibited with IL6 neutralizing antibodies^62^. In myocardial tissue of rats, the IL6/STAT2 pathway was observed to be suppressed and ET-1 levels decreased after treatment with atorvastatin leading to prevention of myocardial fibrosis^63^. In a study of pulmonary hypertension using a heart failure with reduced ejection fraction (HFrEF) model, a first-in-class anti-fibrotic, anti-inflammatory, and anti-proliferative compound PBI-4050 reduced activation of lung fibroblasts by IL6, TGFβ, and ET-1, all three factors found to be upregulated in our study, resulting in reduced lung fibrosis and remodeling^64^. Exposure to other inflammatory cytokines such as tumor necrosis factor-alpha (TNF-α) or interleukin-1 beta (IL-1β) have induced transformation of human DMVECs into myofibroblasts^65^. Although the physiology and cell phenotypes may not be the same in regards to burn HTS, these studies provide some insight into cellular mechanisms involved in fibrosis.

Both ANGPT1 and ANGPT2 were found to be secreted by HTS DMVECs. ANGPT1 activation of its receptor tyrosine kinase TIE-2, present primarily on vascular endothelium, promotes quiescence and structural integrity of the vasculature^66^. ANGPT1 has anti-inflammatory effects on the vasculature which is mediated by inhibition of nuclear factor kappa B (NF-kB)^67^, and is capable of decreasing vessel permeability and plasma leakage^68^. Inflammatory stress-induced release of ANGPT2 competes with ANGPT1 for TIE-2 and leads to vascular destabilization, making the endothelium prone to VEGF and subsequent angiogenesis^44,69^. ANGPT2 is stored in and rapidly released upon stimulation from endothelial cell Weibel-Palade bodies^70^. It has been shown to play a role in induction of inflammation by sensitizing endothelial cells to TNF-α and aiding in the proinflammatory response by promoting adhesion of leukocytes to the endothelium^71^.

Based on PCR array results, gene expression for ANGPT2 in HTS DMVECs reached a 21.29-fold increase (average of 13.50) compared to NS DMVECs, this is in comparison to a 5.40 average fold increase of ANGPT1 in HTS DMVECs vs NS. This ANGPT1/ANGPT2 ratio favored toward ANGPT2 likely promotes destabilization of vasculature, vessel remodeling and a proinflammatory state. A previous study that evaluated hypertrophic sternotomy scars demonstrated a decrease in the ANGPT1/ANGPT2 ratio over a year long period, along with higher microvessel density in the scars^44^. In a two year study of mastectomy scars, there was increased expression of VEGF, ANGPT1, ANGPT2, and TIE-2 in fibroblasts/myofibroflasts from early scars and decreased in older scar, while vascular expression of ANG was decreased, ANGPT2 remained constant, and TIE-2 vascular expression increased^72^. The pro-angiogenic environment produced by ANGPT is opposed by the decreased expression of ANG observed in HTS DMVECs.

ANG is a potent mediator of new blood vessel formation. Our findings show that ANG gene and protein expression was similar or decreased in scar DMVECs compared to normal skin DMVECs. Treatment of scar fibroblasts with recombinant ANG has shown to decrease proliferation of human scar fibroblasts and TGFβ1 secretion^73^. An increase in ANG secretion may be necessary for a proper angiogenic and wound healing response, which is altered in HTS after burn injury.

In terms of other down regulated genes, Adhesion G Protein-coupled receptor B1 (ADGRB1) gene had the highest fold downregulation, but its role in wound healing has not been previously described. ADGRB1 encodes for brain-specific angiogenesis inhibitor 1 (BA1) which has an anti-angiogenic role and is downregulated in glioblastoma and metastatic colorectal cancer^74,75^. The downregulation of ADGFRB1 in DMVECs in part supports a pro-angiogenic response.

Confirmatory qRT-PCR without subsequent ELISA was performed for multiple genes identified to be differentially expressed between scar and skin DMVECs when using the PCR array. TIMP3 and PGF gene expression was found to be downregulated based on the microarray but upregulated based on qRT-PCR analysis. MMP2 gene expression was downregulated based on PCR array analysis but inconclusive with mixed results using qRT-PCR. MMP’s are involved in ECM remodeling during wound healing and are increased in scarless fetal wounds relative to TIMPs, whereas the reverse is observed in HTS^76^. TIMPs inhibit MMPs in a 1:1 ratio, and TIMP3 is known to be expressed by endothelial cells, macrophage-like cells, keratinocytes, and fibroblasts^77^. Increased TIMP3 relative to MMP2 would suggest contribution of DMVECs to decreased collagen degradation in HTS. PGF is part of the VEGF family, and functions in synergy with VEGF to promote angiogenesis. PGF knockout mice are observed to have impaired wound healing due to defective angiogenesis^78^. Elevated PGF secretion by DMVECs is consistent with a pro-angiogenic environment in HTS. FIGF gene expression, which encodes for VEGF-D, was not found to be differentially expressed in the PCR array but qRT-PCR analysis found it to be downregulated in scar vs skin DMVECs. As mentioned previously, both PCR array and confirmatory qRT-PCR for TGFβ2 expression showed upregulation.

DMVECs appear to have a mixed expression profile of genes and proteins that, based on previous evidence, can have either contributing or inhibitory effects on the formation and maintenance of HTS. Factors related to a pro-angiogenic response are found to be upregulated by DMVECs, but does not include VEGF, which is known to not be expressed by ECs but is commonly associated with angiogenesis in general. Not directly measured in this pig model were levels of possible endotheliopathy after injury through the use of markers such as SDC-1^9^, but we do illustrate the presence of abnormal endothelial cells in HTS which is evidence for a persistent state of endotheliopathy after burn injury; therefore, further correlation between acute endotheliopathy and HTS remains a potential area of future work.

Such endotheliopathy has been investigated in the setting of acute resuscitation after burn injury. We have previously shown mitigated endothelial cell injury after acute burn injury with the use of fresh frozen plasma in a mouse model^10^. Additional evidence of a persistent hypermetabolic and hyperinflammatory state in pediatric patients up to three years after severe burn injury suggests the potential for long term persistence of endothelial dysfunction^79,80^. Earlier protection of the endothelium against injury or earlier improvement in its function may be helpful in preventing subsequent HTS. Endothelial cell targeted therapy may also be a potential method to treat HTS, whether that be addressing endotheliopathy early or throughout post-burn care, or supplementing current treatments with drugs targeting specific molecular pathways.

This study was limited by the use of technical replicates (n = 6) of NS DMVECs derived from one area of normal skin from one animal in qRT-PCR and ELISA analysis. These confirmation studies were only used on samples from pigs that sustained excisional wounds. The use of different methods of injury may contribute to differences in HTS formation and DMVEC function, but scar characteristics appeared to be similar between the two types of injury in this study. Also, ELISA and qRT-PCR results from excisional HTS DMVECs confirmed findings from PCR array results from burn HTS DMVECs. Burn HTS and excisional HTS were therefore discussed as one entity. Both scar and normal skin samples came from pigs that sustained burn or excisional injuries, therefore due to an initiation of a systemic response in injured pigs that affect DMVECs in non-injured areas, there may be potential of seeing greater differences in gene and protein expression if normal skin samples were taken from uninjured pigs. Comparison of HTS DMVECs to DMVECs from normal scarring would also be useful for further characterization. All biopsies were taken 70–86 days post injury therefore allowing evaluation of a dynamic process only at a single point in time. Due to the potential for changes to DMVEC transcription when changing from the in vivo to the in-vitro environment, low passage cells were used in an attempt to recapitulate the HTS milieu in vivo. However, these cells were cultured over a period of days to weeks prior to assay. Therefore, a survey of gene and protein expression from DMVECs isolated directly from cells without in vitro manipulations is also warranted in future work. Additionally, in future work, the co-culturing of DMVECs with other cell types is necessary and will be pursued to gain more understanding of DMVECs’ involvement in the angiogenic process.

## Conclusions

The findings from this study establish a preliminary functional and expression characterization of dermal microvascular endothelial cells in hypertrophic burn scar. Functionally, HTS DMVECs have increased barrier function and decreased permeability. The profile of DMVEC gene and protein expression consists of factors that are known to potentially propagate the formation of abnormal scar. Factors secreted by active and dysfunctional endothelial cells can interact with surrounding cells in the wound environment to further contribute to HTS. Overall, gene and protein expression leads to cellular function. Cellular function contributes to tissue phenotype which ultimately contributes to symptoms associated with HTS. Further research on the role of endothelial cells in HTS can help characterize complex interactions between cells in future work and lead to more targeted therapies in the treatment of HTS.

## Supplementary Information

Supplementary Information.

## Data Availability

All data generated or analysed during this study are included in this published article [and its supplementary information files].
